# 

**Published:** 1867-07

**Authors:** 


### CONTENTS FOR JULY.

ORIGINAL CONTRIBUTIONS.

Report on Cholera in Chicago in 1866. By W. R. Marsh, 289
Epilepsy Treated by Lactate of Zinc. By C. F. Hart M.D., Chicago, 299
Three Surgical Cases. Read before the Chicago Medical Society. By Hiram Wanzer, M.D., Chicago, 301
The Endoscope, and its Application to the Diagnosis and Treatment of Urinary Affections. By A. J. Desormeaux, M.D., Translated by R. P. Hunt, M.D. Continued from Page 288 337

PROCEEDINGS OF SOCIETIES.

Chicago Medical Society.—Fibroid Polypus of the Uterus.—Cancerous Disease of the Stomach and Liver.—Glaucomatous Eye.—Large Concretion, nearly Filling the Globe, 305–7
Seventeenth Annual Meeting of the Illinois State Medical Society, 308
Military Tract Medical Association, 326
Allen County, Indiana, Medical Association, 329

EDITORIAL.

Supplement, 332
Delay, 332
Communications, 332
Gayetty Outdone, 332
Astronomical, 333
Reducing the Temperature, 333
Scintillations of Similia, 333
The Corner-Stone of Rush Medical College 334
Bellevue Place, 334
Hæmorrhoidal Affections, 334
To Graduates of Rush Medical College, 334
Receipts Acknowledged, 335
Married, 335
Mortality Report for the Month of May, 336

